# Development of a next-generation NIL library in *Arabidopsis thaliana* for dissecting complex traits

**DOI:** 10.1186/1471-2164-14-655

**Published:** 2013-09-25

**Authors:** Richard S Fletcher, Jack L Mullen, Seth Yoder, William L Bauerle, Gretchen Reuning, Saunak Sen, Eli Meyer, Thomas E Juenger, John K McKay

**Affiliations:** 1 Department of Bioagricultural Sciences & Pest Management, Colorado State University, 80523 Fort Collins, CO, USA; 2 Cargill Specialty Seeds & Oils, 80525 Fort Collins, CO, USA; 3 Department of Horticulture and Landscape Architecture, Colorado State University, F80523 Fort Collins, CO, USA; 4 Department of Epidemiology and Biostatistics, University of California San Francisco, 94143 San Francisco, CA, USA; 5 Department of Zoology, Oregon State University Corvallis, 97331 Corvallis, OR, USA; 6 Section of Integrative Biology & Institute of Cellular and Molecular Biology, University of Texas, 78712 Austin, TX, USA

**Keywords:** 2b-RAD, Fine-mapping, Quantitative trait loci, Stomatal conductance

## Abstract

**Background:**

The identification of the loci and specific alleles underlying variation in quantitative traits is an important goal for evolutionary biologists and breeders. Despite major advancements in genomics technology, moving from QTL to causal alleles remains a major challenge in genetics research. Near-isogenic lines are the ideal raw material for QTL validation, refinement of QTL location and, ultimately, gene discovery.

**Results:**

In this study, a population of 75 *Arabidopsis thaliana* near-isogenic lines was developed from an existing recombinant inbred line (RIL) population derived from a cross between physiologically divergent accessions Kas-1 and Tsu-1. First, a novel algorithm was developed to utilize genome-wide marker data in selecting RILs fully isogenic to Kas-1 for a single chromosome. Seven such RILs were used in 2 generations of crossing to Tsu-1 to create BC1 seed. BC1 plants were genotyped with SSR markers so that lines could be selected that carried Kas-1 introgressions, resulting in a population carrying chromosomal introgressions spanning the genome. BC1 lines were genotyped with 48 genome-wide SSRs to identify lines with a targeted Kas-1 introgression and the fewest genomic introgressions elsewhere. 75 such lines were selected and genotyped at an additional 41 SNP loci and another 930 tags using 2b-RAD genotyping by sequencing. The final population carried an average of 1.35 homozygous and 2.49 heterozygous introgressions per line with average introgression sizes of 5.32 and 5.16 Mb, respectively. In a simple case study, we demonstrate the advantage of maintaining heterozygotes in our library whereby fine-mapping efforts are conducted simply by self-pollination. Crossovers in the heterozygous interval during this single selfing generation break the introgression into smaller, homozygous fragments (sub-NILs). Additionally, we utilize a homozygous NIL for validation of a QTL underlying stomatal conductance, a low heritability trait.

**Conclusions:**

The present results introduce a new and valuable resource to the Brassicaceae research community that enables rapid fine-mapping of candidate loci in parallel with QTL validation. These attributes along with dense marker coverage and genome-wide chromosomal introgressions make this population an ideal starting point for discovery of genes underlying important complex traits of agricultural and ecological significance.

## Background

Linkage mapping of QTL is a common statistical approach in plant genetics where recombinant populations generated from crosses between inbred parent lines are used, in combination with molecular markers, to identify loci associated with variation in continuously distributed traits [1-8]. Mapping populations common to QTL analyses are many and include doubled haploids (DH), F2, backcross, advanced intercross, nested association mapping and RILs. Mapping QTL for complex traits is now routine, with the typical output being QTL spanning large confidence intervals encompassing many (hundreds or more) possible causal genes [9].

The steps following QTL identification frequently involve functional validation of the QTL, and refinement of location (fine-mapping) towards the goal of identification of a causal gene – the major challenge in quantitative genetics today [10]. One of the most common approaches for accomplishing these objectives is through the development and phenotypic characterization of NILs [11]. The generation and phenotyping of NILs is considered a laborious and time consuming process, but the robust design leads to a minimal false positive rate.

NILs are lines containing a single or small number of genomic introgressions from a donor parent in a different and otherwise homogeneous genomic background. By homogenizing all genetic factors outside of the focal genomic region, the true effect of the QTL on the phenotype can be estimated relative to the line into which the introgression was introduced (i.e. void of the chromosomal introgression) [12]. In addition to the simplification of genetic analyses, NILs are considered genetically 'immortal’ [13] which allows for replicated experiments across multiple environments resulting in more accurate estimates of effect size for complex traits. NILs have proven to be an effective resource for QTL validation and a logical starting point for the creation of fine-mapping populations [14-21].

Creation of a single near-isogenic line generally starts by crossing a line carrying the targeted QTL region to one of the parental lines of the population, thus creating a backcross population. Genome-wide genotyping of the backcross progeny is performed to identify recombination events allowing for selection of progeny which carry the target chromosomal introgression derived from the donor and recurrent parent genome elsewhere. Subsequent generations of self-pollination (selfing) are normally required to achieve homozygosity of the introgressed region and the process can take several backcrossing cycles to produce a NIL carrying an introgression of acceptable size and genomic location. An alternative approach has been the use of heterogeneous inbred families (HIFs) where NILs are selected from incompletely inbred lines which still harbour a small amount of heterozygosity at random intervals across the genome [22,23]. Analysis of a HIF population with molecular markers allows for the selection of lines heterozygous at a candidate genomic location, which in combination with further selfing and genotyping, enables selection of NILs derived from several heterogeneous genetic backgrounds. Producing NILs with smaller introgressions requires greater effort. Large populations are needed to break up small chromosomal segments, and high-density genotyping is required to discover them.

A NIL library is a family of near-isogenic lines where each line carries a different donor parent fragment and the population carries introgressions spanning the entire genome [24]. A NIL library is an ideal starting point for QTL validation, especially in cases where the library is derived from parent lines for which an immortal recombinant population (i.e. RILs, DH, etc.) already exists. In this case, QTL identified via traditional linkage mapping experiments performed on the mapping population can be immediately tested by selecting NIL(s) representing the QTL introgression and testing them for a phenotypic effect relative to the wild type recurrent parent. NIL libraries are also valuable starting material for fine-mapping QTL through the creation of sub-NILs [25], recombinant lines in which the original NIL introgression is broken into smaller genomic fragments. In this case, a candidate NIL is backcrossed to the recurrent parent and the progeny are genotyped using markers specific to the introgression region so that individuals carrying genomic fragments spanning the length of the original introgression can be identified. Subsequent phenotyping of the sub-NILs provides finer resolution of the region controlling the trait of interest, effectively narrowing the list of possible causal genes.

Several NIL populations are currently available to the Arabidopsis research community. Koumproglou *et al*. [26], using 31 simple sequence repeat (SSR) markers, created a population of Chromosome Substitution Strains by replacing chromosomes from the accession Columbia (Col-0) with homologous chromosomes from the accessions Landsberg *erecta* (L*er*) and Niederzenz (Nd). Additionally, a population of more traditional NILs were created in a systematic approach where increasing lengths of chromosomal introgressions were introduced from L*er* into the Col-0 background. Keurentjes *et al*. [27] generated a population of 92 NILs carrying genome-wide chromosomal introgression from the accession Cape Verde Islands (Cvi) into the L*er* background. Selections were made from the genotyped RIL mapping population described by Alonso-Blanco *et al*. [28] and used in backcrosses to create the NIL library. The RIL population has been mapped for QTL underlying flowering time and carbon isotope ratio (*δ*^13^C) [29], recombination frequency [30], seed germination [31], seed mineral concentration [32] and fructose sensitivity [33]. The same 321 AFLP (Amplified Fragment Length Polymorphism) markers used to build the RIL map were used in the NIL breeding scheme. Finally, Torjek *et al*. [34] created a population of 140 reciprocal NILs from the accessions Col-0 and C24 (78 NILs in the Col-0 background and 62 lines in the C24 background) utilizing a total of 125 markers [35]. This NIL library has been used in subsequent studies of epistasis [36] and heterosis [37].

Here we report the development of a new population of 75 NILs constituting genome-wide chromosomal introgressions. The NIL population exploited inbred lines selected from the RIL population described in McKay *et al*. [38] as the starting material for backcrossing. Briefly, the RIL population is derived from a cross between the *A*. *thaliana* ecotypes Tsu-1 (CS1640), an accession originating from Tsushima, Japan and Kas-1 (CS903), an accession originating from Kashmir. These sites of collection are among the wettest and driest habitats, respectively, in the *A*. *thaliana* species range and the accessions differ in several aspects of drought physiology [39,40]. Recombinant populations derived from these diverse accessions will therefore segregate alleles underlying variation in these physiological traits, providing a powerful resource for identifying functional genes.

We developed a population of 75 *Arabidopsis thaliana* NILs containing both homozygous and heterozygous introgressions, enabling simultaneous pursuit of QTL validation and fine-mapping. Genotyping the population with over 1,000 molecular markers has provided us with excellent resolution on the total number of introgressions existing in each NIL as well as their location and length. It is the most densely genotyped NIL population developed thus far by more than 3-fold. The utility of the NIL library is demonstrated in a simple case study where, in a single generation, we utilize a homozygous NIL to validate and localize a QTL for a low heritability physiological trait (g_0_; night-time stomatal conductance) while concurrently selfing heterozygous selections to create sub-NILs for further fine-mapping.

## Results

### Marker-assisted NIL breeding program

**Figure 1:**
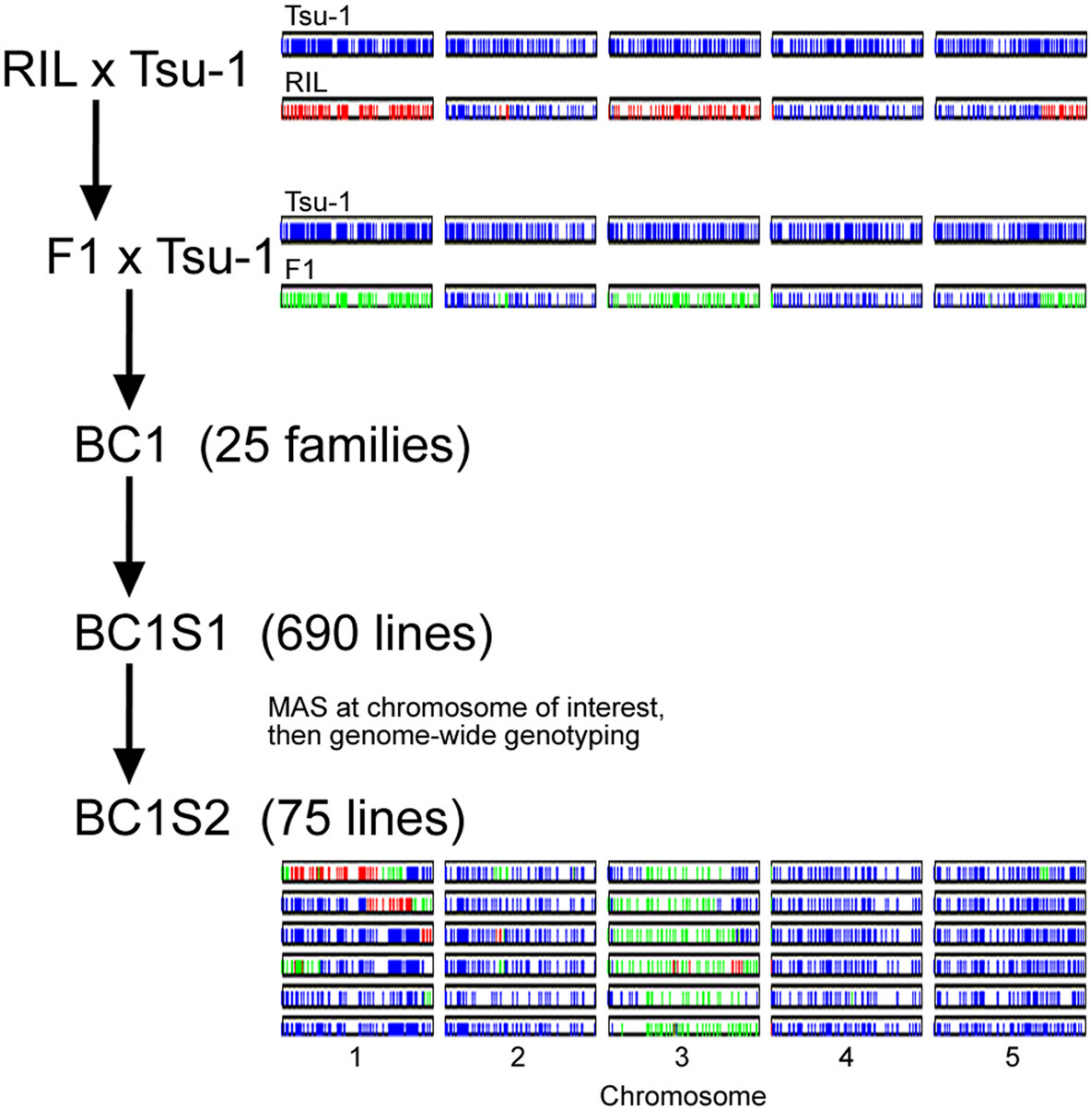
**Breeding scheme of the NIL library.** Breeding scheme and graphical genotypes of a set of NILs containing both homozygous introgressions (Chromosome 1) and heterozygous introgressions (Chromosome 3) derived from a single RIL. Each diploid breeding line is represented by a single row of 5 chromosomes where red coloring represents Kas-1 genotypes; Blue, Tsu-1; Green, heterozygous. Graphical genotypes of 6 of the 75 lines are shown.

Figure 1 shows the breeding design for the NIL library. An algorithm was developed [see Additional file 1] to select RILs homozygous for Kas-1 alleles across one of each of the 5 *Arabidopsis* chromosomes. The results found 7 such RILs from the population of 346. These RILs were crossed to Tsu-1 and progeny were genotyped to confirm they were truly F1s. These were then crossed back to Tsu-1, creating 25 BC1 families. Plants from each BC1 family were genotyped at the chromosome of interest to select individuals carrying Kas-1 alleles so they could be self-pollinated to generate BC1S1 seed. BC1S1 plants were genotyped using 48 genome-wide SSRs described in McKay *et al*. [38]. These data were analyzed using an algorithm [see Additional file 2] designed to identify a subset of lines representing Kas-1 chromosomal introgressions spanning the genome in otherwise Tsu-1 backgrounds. The algorithm was used to select 103 BC1S1 plants which were screened at an additional 149 single nucleotide polymorphisms (SNPs) loci using the Sequenom MassARRAY® (Sequenom, San Diego, CA). Only 41 of 149 SNPs were informative for the parental lines. Finally, an additional 930 polymorphic loci were revealed using 2b-RAD [41] whereby genome complexity is reduced using class IIB restriction enzymes followed by sequencing on the SOLiD platform (Applied Biosystems, Foster City, CA).

### Polymorphisms detected between Tsu-1 and Kas-1 by 2b-RAD genotyping

Restriction site–associated DNA (RAD) tag sequencing reduces genome complexity by focusing only on DNA flanking the recognition sites of the selected restriction endonuclease [42]. The RAD method used in this study, described in [41] is a simple and effective means of discovering a large number of SNPs unique to the study population, avoiding the ascertainment bias associated with SNPs discovered via population surveys [43]. The 2b-RAD method utilizes the type IIB restriction enzyme, *AlfI*, which operates by cleaving DNA both upstream and downstream of the recognition site. The resulting tags are uniform in length, making them ideal for amplification and sequencing on next-generation platforms. Following digestion, tags were labelled with sample-specific oligonucleotide barcodes for multiplexed sequencing. Finally, reads were quality filtered and aligned to a collection of *AlfI* sites in the Col-0 Arabidopsis reference genome (TAIR9) in order to assign a physical location to each SNP.

**Figure 2:**
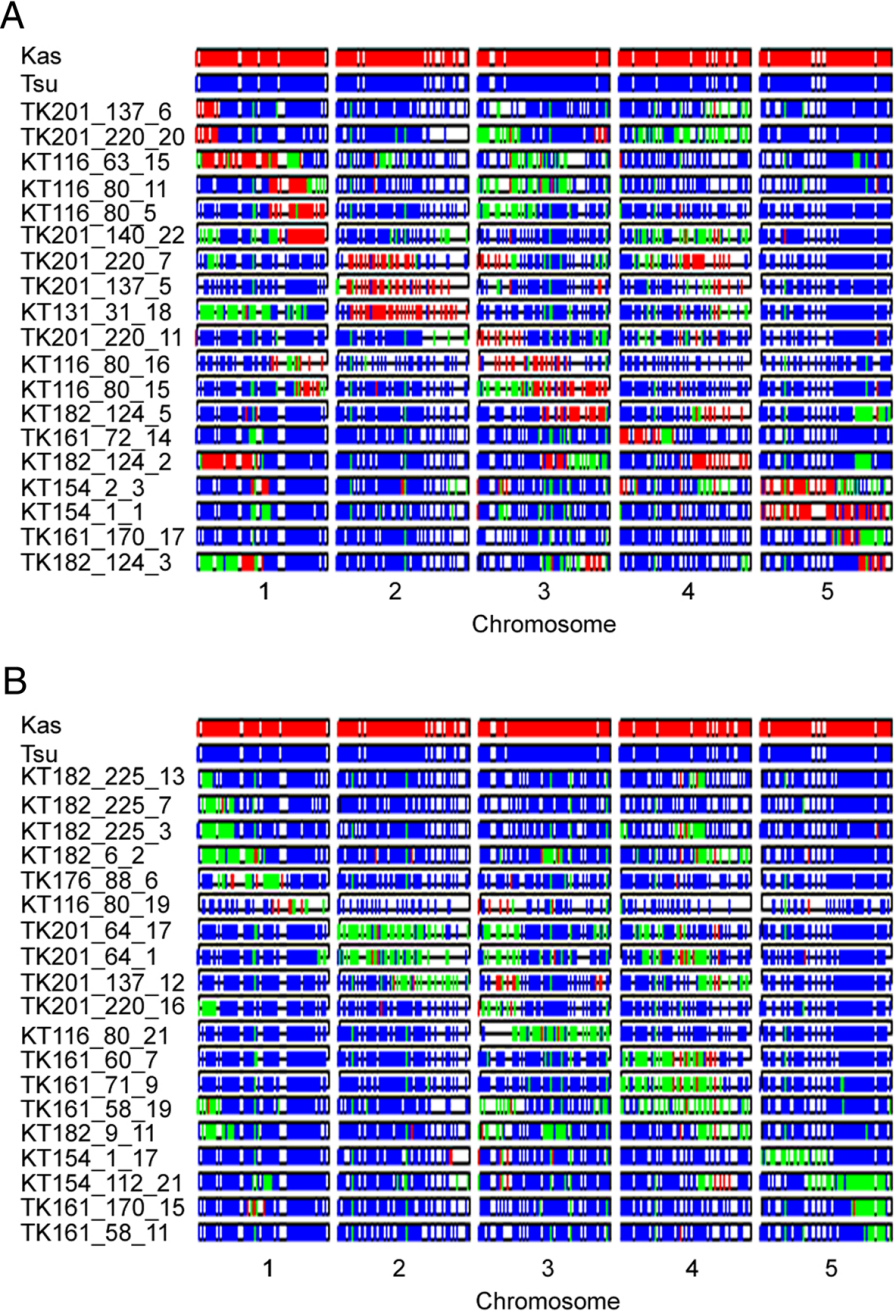
**Graphical genotypes of NILs representing (A) homozygous and (B) heterozygous introgressions cumulatively spanning the length of the genome.** Red, Kas-1; Blue, Tsu-1; Green, heterozygous.

Initially, 1319 polymorphisms were identified between the parent lines Tsu-1 and Kas-1 based on the 2b-RAD tags that were sequenced. Because these NILs are derived from a known pedigree of previously genotyped individuals, we were able to filter to include SNPs that would segregate in the progeny, resulting in a final set of 930 loci with high-confidence genotypes for use in subsequent population analyses. A non-trivial fraction of markers remained as missing data in each sample due to the stringent scoring criteria of our method. The majority of uncalled loci in typical 2b-RAD datasets are discarded because of low coverage (Meyer, unpublished observations) so this problem could be mitigated with deeper sequencing. However, the known pedigree of these samples and the low level of recombination made it possible to accurately reconstruct haplotypes despite these missing data. The filtered data were used to construct graphical genotypes [44] of the NIL population, a subset of which are represented in Figure 2. We also provide a database of the genotypes for the entire NIL population [see Additional file 3]. In addition, both parental accessions have been re-sequenced and the genome-wide reads have been deposited in the Short Read Archive (http://www.ncbi.nlm.nih.gov/Traces/sra/sra.cgi) and posted on the 1001 Genomes Project website (http://www.1001genomes.org/) so the details of the 930 SNPs utilized in this study can be accessed at these resources.

### Genomics of chromosomal introgressions in the NIL population and the added value of increased marker resolution

**Figure 3:**
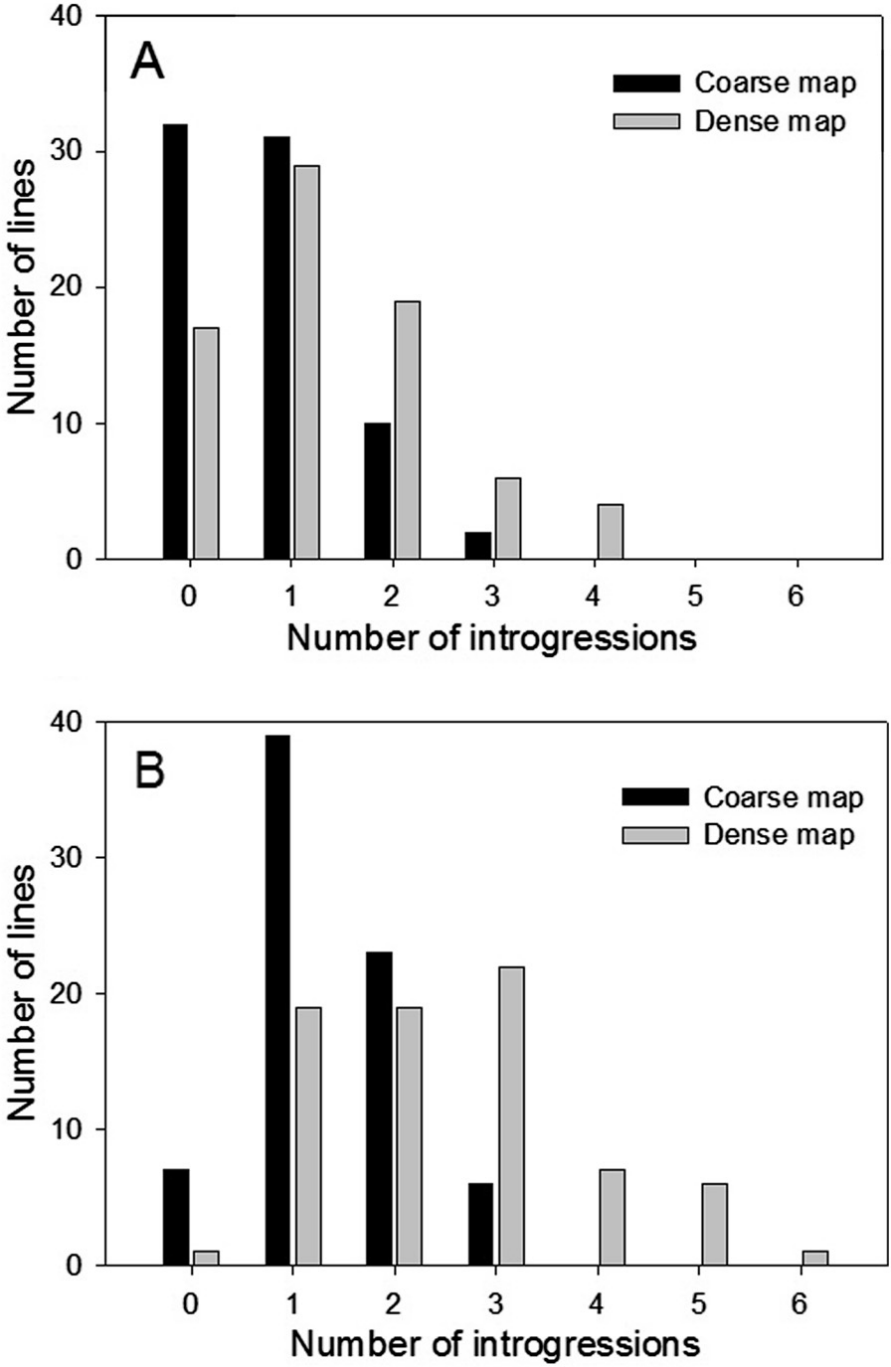
Distribution of (A) homozygous and (B) heterozygous introgression number, estimated using the coarse and dense maps.

Across the 75 NILs, the average number of homozygous introgressions per NIL was 1.35 and ranged from 0 to 4 while the average number of heterozygous introgressions was 2.49 and ranged from 0 to 6 (Figure 3). The average number of introgressions per chromosome was 57.6, ranging from 34 on chromosome 2 to 79 on chromosome 1 [see Additional file 4]. The total length of homozygous introgressions was 506 Mb compared to nearly 949 Mb of heterozygous chromosomal introgression which represent 4.3 and 8.0 times the total length of the Arabidopsis genome, respectively. Together these results suggest we have reached our goal- the entire genome is represented as a Kas-1 introgression for each genotypic state (i.e. zygosity) in at least one NIL, thus enabling QTL validation and fine-mapping for any locus of interest.

**Figure 4:**
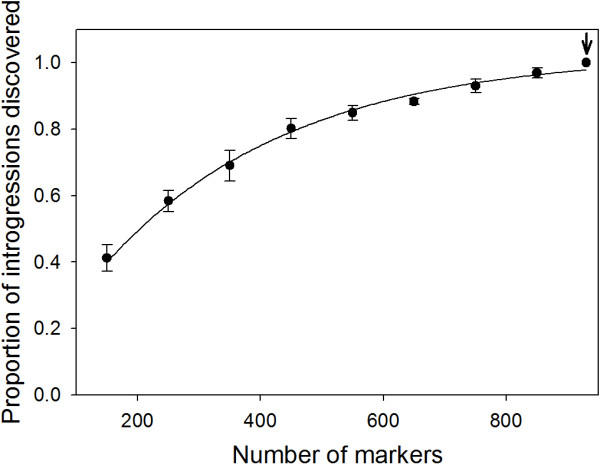
**Heterozygous introgressions discovered at varying marker densities.** Estimates are based on 10 repetitions of markers selected at random from the dense map for each marker density (mean ± SD). The line is fitted based on an exponential rise function. The data point marked by an arrow represents the final estimates generated from the dense map. The plot of homozygous introgression discovery was nearly identical and excluded to simplify the figure.

The additional loci accounted for by 2b-RAD genotyping resulted in a final marker density of 2.24 markers per cM, based on the estimated 450.8 cM map of the Kas-1 × Tsu-1 RIL population. This is a significant improvement in resolution from the 0.18 markers per cM when using only the original SSR and Sequenom marker set (hereafter referred to as the coarse map). In spite of the high frequency of uncalled alleles, 128 new introgressions were revealed which summed to nearly 539 Mb of DNA (164 Mb homozygous and 375 Mb heterozygous) that would have been missed without the additional markers from 2b-RAD genotyping. To illustrate this effect we re-sampled the dataset at varying marker densities (Figure 4). The exponential curve fit (r^2^ = 0.99) used to estimate introgression detection begins to level above 800 markers, suggesting diminishing introgression discovery with more extensive genotyping. In a comparison of the NILs using the coarse map relative to the dense map created from 930 2b-RAD markers, the average size of a homozygous introgression in the coarse map was 18% larger (1.2 Mb) than in the dense map. Similarly, the average heterozygous introgression size in the coarse map was 19% larger (1.3 Mb), confirming that the additional markers were identifying smaller introgressions missed in the coarse map. This fact is highlighted by the total number of introgressions [see Additional file 4] discovered using the denser marker set. The result was a 1.8-fold (Figure 3) increase in the number of homozygous and heterozygous introgressions discovered.

### Case study: utilizing selections from the NIL library for QTL validation and sub-NIL development

To demonstrate the value of this new resource, we analyzed the RIL population [38] for QTL for night-time leaf conductance (g_0_). g_0_ is a low-heritability, quantitative trait that is important for plant-water relations and mineral nutrition. While the adaptive value of g_0_ has yet to be fully understood, incomplete stomatal closure during the night can lead to substantial transpirational water loss [45]. Variation in this trait has been found among and within species, and it correlates with some daytime gas-exchange traits such as water-use efficiency (the ratio of CO_2_ assimilation to transpiration) [46]. Estimates of transpiration have been found to be particularly sensitive to g_0_[47], making it an interesting candidate for studies on the physiology and genetics of plant drought adaptation. In view of that, intraspecific variation in observed g_0_ has been found to have the largest effect on transpiration across a species’ native habitat (Bauerle, unpublished observations).

**Figure 5:**
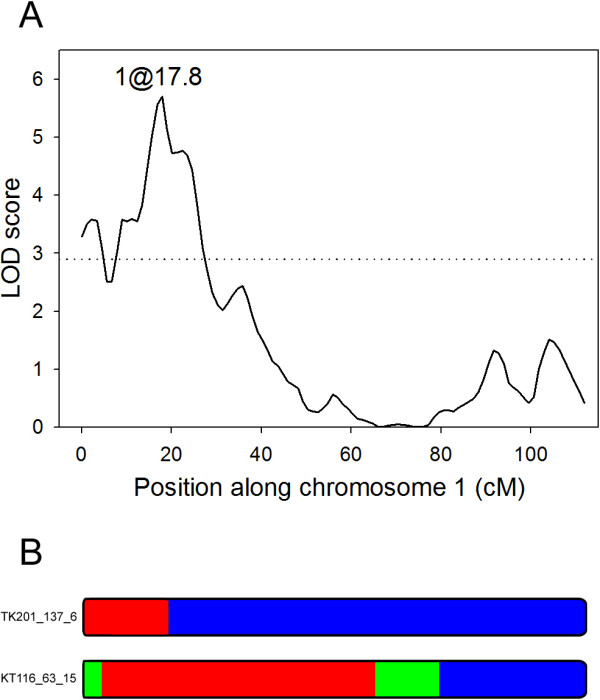
**QTL location and graphical genotypes of the NILs used in the QTL validation. (A)** Localization of the dark-conductance QTL along chromosome 1, the dotted line indicates the threshold LOD score. **(B)** Graphical genotypes scaled to represent the genetic distance (cM) of the x-axis of panel A of chromosome 1 for the NILs used in the QTL validation.

Significant variation in night-time conductance was observed among the RILs. We identified a single QTL for g_0_ on the top of chromosome 1 (Figure 5A), which explained 9% of the variance in g_0_, and found the trait to have relatively low broad sense heritability (H^2^ = 0.21) in this population. Lines having Kas-1 alleles of markers at the QTL had lower dark conductance, consistent with the dry habitat of the Kas-1 parent [see Additional file 5]. Additional loci were identified on chromosomes 2 and 4 below the threshold of significance, which may have had marginal effects on g_0_ [see Additional file 6].

To validate the QTL we selected two NILs homozygous for a Kas-1 introgression spanning the QTL and measured g_0_ relative to Tsu-1 with the expectation that one or both would have a significantly lower g_0_ value. NIL TK201_137_6 carries an introgression estimated to span physical positions 505,086 to 5,273,972 on chromosome 1 and KT116_63_15 is estimated to carry a much larger introgression between positions 2,040,091 and 19,225,223 (Figure 5B). KT116_63_15 also carried small heterozygous regions at either end of the homozygous introgression. Large and highly significant differences were found between both NILs and Tsu-1 (Table 1), providing strong evidence for the presence of the QTL and providing a surprisingly high estimate of the relative difference in g_0_ conferred by the two alleles when compared to the results of the initial QTL experiment [see Additional file 5]. The region between 5,273,972 and 19,225,223 can be effectively eliminated from consideration for harbouring the causal locus since TK201_137_6 was significantly different from Tsu-1 and did not carry Kas-1 DNA in this interval [24]. It is worth noting that both NILs carried introgressions on chromosomes other than the chromosome one focal area. However, none of them were common between the NILs and the difference in g_0_ values between KT116_63_15 and TK201_137_6 was non-significant which suggests these introgressions were not impacting our results substantially.

**Table 1:** **Results of QTL validation experiment comparing NIL g**_**0 **_**values with Tsu-1**

**Comparison**	**g**_**0**_	**Standard**	**Difference**	**t value**
	**(mmol m-2 s-1)**	**Error**	**NIL - (Tsu-1)**	
Tsu-1	119.76	11.55	n/a	-0.94
TK201_137_6	52.42	10.99	-67.34 **	-4.02
KT116_63_15	67.45	11.40	-52.30 **	-3.15

Difference values with ** are highly significant (P < 0.0001).

Nearly 1,500 genes are predicted to lie within the region spanning physical positions 505,086 to 5,273,972 of chromosome one. We have assembled a list of candidate genes based upon hits to gene ontology (GO) terms relevant to stomatal conductance: abscisic acid (ABA), stomata and water [see Additional file 7].

**Figure 6:**
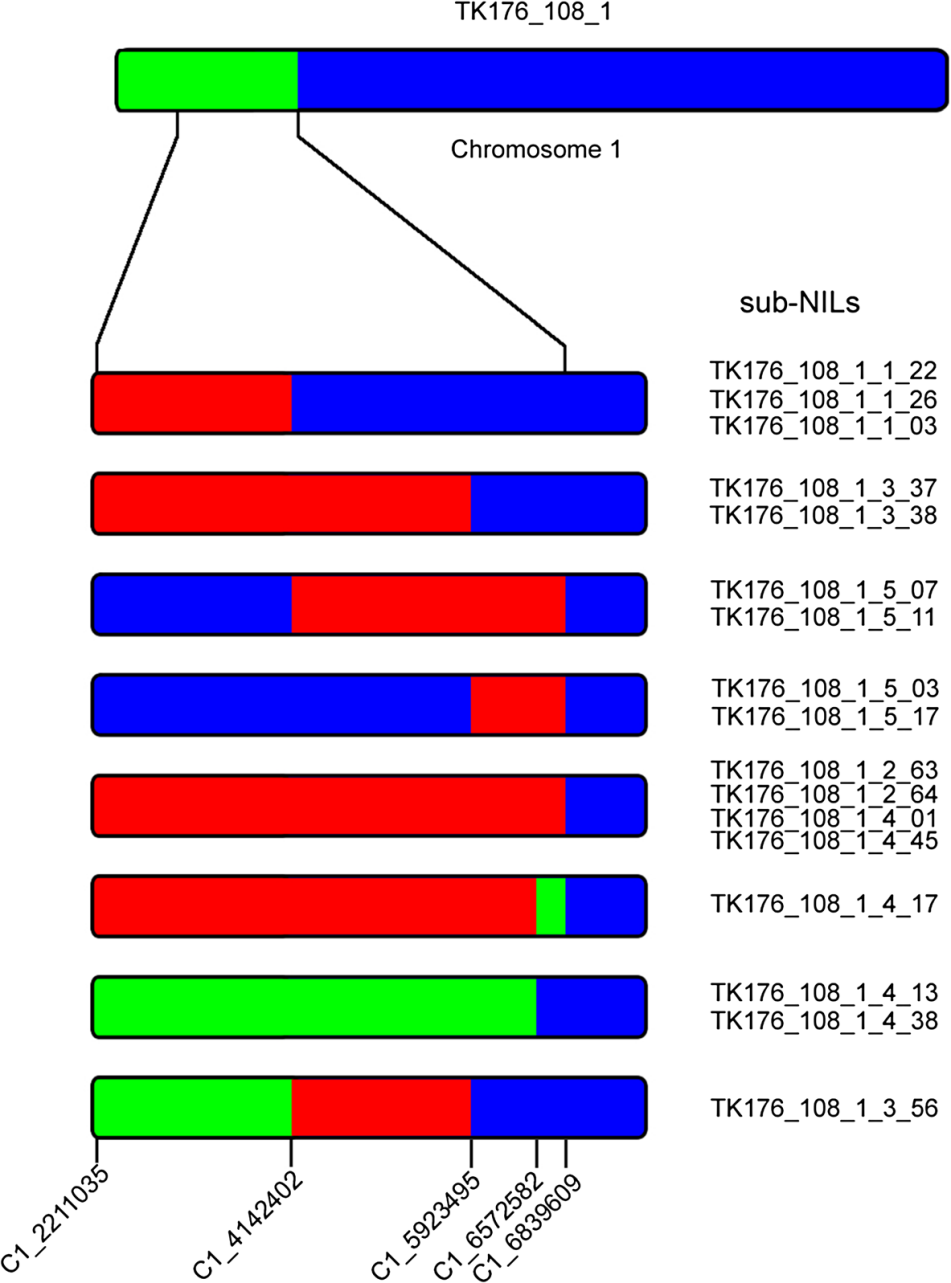
**Diagram of genotype information for fine mapping lines.** Heterozygous NIL selected for selfing **(top)** and the detailed focal region of the selected sub-NILs **(bottom)**. The numbers at the bottom of the sub-NILs indicate the physical position of markers and, therefore, represent estimated introgression boundaries. Red, Kas-1; Blue, Tsu-1; Green, heterozygous.

To illustrate the power of deriving sub-NILs from heterozygote NILs, concurrent to the QTL validation experiment we planted seeds derived from a line heterozygous in the roughly 3 Mb g_0_ QTL interval (Figure 5). We selected 5 polymorphic loci from a panel of validated SNPs described in [38] for genotyping a population of 286 BC1S3 individuals (BC1S1 graphical genotype is represented in Figure 6). The marker representing the lower end of the interval at physical position 6,839,609 did not segregate and all individuals were homozygous for the Tsu-1 allele. The genotype for the 2b-RAD allele near this location was scored as "not genotyped" in the original BC1S1 genotyping so we were unsure exactly where this particular heterozygous introgression ended. In the end, we were left with 4 informative markers in the physical interval spanning positions 2,211,035 to 6,572,582. We selected 17 recombinants (Figure 6) representing the majority of the recombination events possible. Unfortunately, no double recombinants were discovered so that a sub-NIL representing the Kas-1 alleles at the middle of the interval could be recovered. However, heterozygous individuals TK176_108_1_4_13 and TK176_108_1_4_38 were kept for selfing and will be available for re-planting to accomplish this since a crossover has already occurred at the lower end. Ultimately, individuals were recovered in this single selfing generation that could be used in the next generation for g_0_ phenotyping experiments to effectively narrow the QTL interval down to, at most, the 1.9 Mb interval between markers C1_2211035 and C1_4142402, an interval predicted to carry about 590 genes.

## Discussion

### Maintenance of homozygous and heterozygous NILs facilitates simultaneous QTL validation and fine-mapping efforts

Near-isogenic lines remain the ideal starting material for validation of QTL as well as breeding schemes designed for fine-mapping with the end goal being the identification of candidate genes [48-51]. QTL validation is relatively straightforward and consists simply of phenotyping NILs with introgressions at the region of interest for the trait of interest. Creation of a suitable population for fine mapping is not as straightforward and is normally a three-generation process that starts with a cross between an inbred NIL and the recurrent parent. This is typically followed by a generation of self-pollination to allow for recombination in the introgression region. The seed harvested from these self-pollinated plants can then be genotyped with markers specific to the region so that homozygous sub-NILs can be identified. The process is fairly straightforward and inexpensive in the context of physical resource, but there is a time cost of at least 3 generations (equivalent to a minimum of 18 weeks).

Our case study illustrates the advantages of maintaining both homozygotes and heterozygotes in the NIL population, combining the benefits of traditional homozygous NILs with the advantages of HIFs [22,23]. For example, measuring g_0_ on the homozygous NILs provided strong evidence for the presence of the QTL in a single generation, thus avoiding the process of generating homozygous lines that would be necessary in HIF populations. These results provided a better estimate of the QTL effect size relative to the results derived from our QTL mapping approach and have justified further investments in fine-mapping using heterozygous NILs. This emphasizes the power NILs create by isolating the genetic factors controlling a phenotype to a single locus as there were other loci worthy of consideration as contributors to variation in g_0_ in the RIL population. Analysis of the genes predicted to lie within this interval revealed a majority of them had GO annotations related to ABA, the major signalling molecule in stomatal regulation [52-54], but examination of the entire region with the AmiGO enrichment analysis tool [55] found it was not significantly enriched for ABA genes. Inspection of the physical location of these ABA-associated candidates reveals that they are clustered in a 1.2 Mb interval (At 1 physical interval: 712,473-1,894,148) which represents a relatively small portion of the 4.8 Mb introgression tested, thus providing an interesting focal region during fine-mapping of the g_0_ phenotype.

With regards to fine-mapping, selfing a heterozygous NIL selection from the population yielded several sub-NILs suitable for phenotyping or additional genotyping in future generations, an attribute common with HIF populations and advantageous over traditional NILs. This was accomplished using a modest population size of BC1S3 plants (n = 286) and the interval could be narrowed down further through genotyping at a higher number of loci and increasing the population size [56]. Regardless, in a six-week period we have identified a population encompassing recombinants in the 4.8 Mb region identified as causal during the QTL validation experiment, translating to a 3-fold change in total time versus a breeding scheme utilizing inbred NILs.

### 2b-RAD is an efficient method for dense genotyping of recombinant populations

*Arabidopsis thaliana* recently celebrated its 25th anniversary as a model organism and now stands alone as the most thoroughly studied plant species on record http://www.arabidopsis.org/, [57]. Recent efforts are producing comprehensive polymorphism databases (http://www.arabidopsis.org/, http://signal.salk.edu/cgi-bin/AtSFP). To interpret the significance and functional consequences of this natural variation, we need to understand the multivariate phenotypic consequences of these variants. NIL libraries, mutants and complementation studies are the tools required for this mechanistic understanding.

The 2b-RAD method added an additional 930 high confidence genotypes to our map providing a level of resolution not yet achieved in any of the Arabidopsis NIL populations described to date. The value of these additional markers is obvious as we compare the coarse and dense maps. The discovery of an additional 129 introgressions is clearly important when making selections for QTL validation. For instance, three additional homozygous introgressions were discovered in KT154_2_3, changing the estimate from one to four. This is a clear illustration of the risks associated with utilizing NILs genotyped at low density in experiments aimed at QTL validation. These offsite introgressions may have effects on the phenotype of interest, potentially resulting in erroneous or uncertain conclusions regarding the QTL effect size and location.

### The Kas-1 × Tsu-1 RIL and NIL populations are a valuable resource for research on the genetics of drought adaptation in the Brassicaceae

Substantial variation for several traits relevant to drought adaptation have been observed in the Kas-1 × Tsu-1 RIL population including *δ*^13^C, leaf water content, instantaneous transpiration rate, flowering time, abscisic acid content and root mass [38], unpublished results]. Accordingly, the NIL population described herein is expected to vary for the same traits, providing a powerful resource for moving from QTL, encompassing thousands of genes, discovered in the RIL population towards a smaller list of putative functional candidates.

No other plant species has been more studied or characterized than *Arabidopsis thaliana*[57]. A high degree of sequence collinearity between it and members of the agriculturally significant *Brassica* genus was discovered over a decade ago [58]. Similar levels of synteny have been found in comparisons with other taxa in the Brassicaceae [59-62]. These results suggest that translational genomics, that is utilizing basic research findings in model organisms to answer practical research questions in species of higher economic value or importance [63,64], could be a viable avenue in understanding complex traits. In this regard, we suggest the Kas-1 × Tsu-1 populations as the ideal starting point for basic research on the genetics and genomics of drought adaptation.

## Conclusions

We have developed a population of 75 NILs that provides genetic resources for fine-mapping QTL as well as QTL corroboration. The high marker density used to construct the population provides a level of resolution not yet seen in a NIL population, thus minimizing ambiguity in fine-mapping and QTL validation studies caused by unidentified chromosomal introgressions elsewhere in the genome. The unique variation that exists between the parents used to construct this resource provides a valuable asset for research focused on identifying the genes responsible for drought adaptation in Arabidopsis and beyond.

## Methods

### Plant material & growth conditions

The *A*. *thaliana* accessions Kas-1 (CS903) and Tsu-1 (CS1640) were used as the original parent lines in developing the RIL population of 346 lines. Kas-1 and Tsu-1 were chosen as parents for developing this population as a result of their extreme differences in water use efficiency as measured by δ^13^C [39,40]. RILs from this population served as the starting point for the NIL breeding program described below.

For the QTL experiment, seed of the RILs along with the parents were sown on soil (Fafard 4P mix, Conrad Fafard Inc., Agawam, MA) in 3-inch pots. Seeds were planted in a randomized complete block design consisting of 2 blocks, and then the pots were refrigerated at 4°C in darkness for 5 d to cold-stratify the seeds prior to commencement of a 8:16 h (light: dark) photoperiod in Conviron ATC60 growth chambers (Controlled Environments, Winnipeg, MB), at 23°C and 40% humidity during the day and 20°C and 50% humidity during the dark period. Light intensity was approximately 330 μmol m^-2^ s^-1^. Plants were grown for approximately 6 weeks prior to measurement. Stomatal conductance was measured in darkness on non-senescing leaves that were large enough to fully accommodate the leaf chamber (1 cm × 2 cm), using an infrared gas analyser (model Li-Cor 6400, LiCor Inc., Lincoln, NE). Prior to measurement the plants were dark adapted for 20 – 28 h. A humidifier was used to reduce variation in humidity over the course of the measurements. For each leaf 10 measurements were taken, with an interval of 10 s between measurements.

For the QTL validation experiment, plants were grown in a randomized complete block design consisting of 3 blocks where each genotype was replicated 6 times within each block. Plants were grown under exactly the same conditions as those described above except that the photoperiod was increased to 12;12 h (light:dark) to accommodate other experiments conducted in the same chamber. One major difference between the two experiments was the use of leaf porometers (model SC-1, Decagon Devices, Inc., Pullman, WA) rather than an infrared gas analyser for stomatal conductance estimates. Two non-senescing leaves were measured on each plant following the manufacturer’s recommended protocol.

### Genetic analyses

Broad-sense heritability was estimated by calculating the ratio V_G_:V_P_, where V_G_ is the among-RIL component of variance and V_P_ is the total phenotypic variance. QTL mapping was performed in the R/qtl program of the R statistical package [65,66], using Haley-Knott regression. Significance thresholds were determined using 1000 permutations. A penalized stepwise approach [67] was used for selection of a multiple-QTL model.

For the QTL validation experiment, data were analyzed with a linear mixed model using PROC MIXED in the SAS software package (SAS Institute Inc. 2003, Cary, NC) where block, row and column effects were treated as random.

### Marker assisted NIL breeding program

To start, 7 RILs were selected from the original population of 346 using the code supplied in an additional file [see Additional file 1]. These 7 represented lines homozygous for Kas-1 alleles across one of each of the 5 chromosomes and all were crossed to Tsu-1 at least 10 times. Some attempted crosses may result in self-pollination due to technical error, thus we genotyped progeny to confirm they were F1s. In general, the real F1s were several times larger than the midparent value, so genotyping was almost unnecessary. Confirmed F1s were crossed back to Tsu-1 and each fruit was collected separately and considered a BC1 family, ultimately creating 25 families. 24 plants from each family were genotyped at the chromosome of interest and selected for selfing to generate BC1S1 seed. In addition to culling the occasional plant generated due to self-pollination, it was also necessary to remove individuals sired by (haploid) pollen from the F1 carrying Tsu-1 alleles for the chromosome of interest. In the next generation, 690 BC1S1 plants were genotyped with the 48 genome-wide SSRs described in [38]. These were then ranked using an algorithm [see Additional file 2] to find lines that were largely Tsu-1, but carrying Kas-1 introgressions spanning the genome. In the end, 75 lines were selected which we screened at an additional 149 loci using the Sequenom MassARRAY® platform, of which 41 were polymorphic. 930 polymorphic loci were added to this marker data set via 2b-RAD [41] where class IIB restriction enzymes are used minimize genome complexity for a final total of 1011 genotyped.

### DNA extraction and genotyping

Genomic DNA was isolated from lyophilized tissue collected from approximately 4-week-old, chamber grown plants using the DNeasy Plant Mini Kit (Qiagen, Valencia, CA) according to the manufacturer’s instructions.

The 48 polymorphic microsatellites used in this study were selected from the large number of those available in *A*. *thaliana*[68,69], arabidopsis.org] due to easily distinguishable allele calls. Descriptions of the primers, PCR conditions and allele scoring are explained in [38].

DNA samples were used to prepare 2b-RAD libraries as previously described [41]. A detailed protocol is available at the Meyer laboratory website (http://people.oregonstate.edu/~meyere/). Briefly, library preparation for 2b-RAD genotyping began with digestion of gDNA samples with *AlfI* (Fermentas) for 37°C for 3 h followed by ligation of adaptors at 4°C for 16 h. Ligation products were amplified by PCR and barcodes introduced to gel-extracted products in a second PCR reaction. Finally, libraries were pooled for multiplex sequencing on the SOLiD sequencing platform (Applied Biosystems). Raw sequences were processed to exclude low-quality reads, and the HQ reads that remained aligned in color-space using the SHRiMP software package [70] to *AlfI* sites extracted from the Arabidopsis genome (TAIR9). A custom Perl script was applied to eliminate short, statistically weak and ambiguous alignments (reads matching multiple sites equally well). Finally, genotypes were determined from nucleotide frequencies using custom Perl scripts to classify each locus as homozygous (minor allele frequencies [MAF] <1%), heterozygous (MAF > 25%), or undetermined (1% > MAF >25%). 20× coverage was required in the parental genomes to identify these alleles with high confidence, and a relaxed threshold of 10× in all other samples to maximize marker densities. Each polymorphic locus identified in these genotypes was compared with the parental genotypes (Tsu-1 and Kas-1) to assign it to one of these backgrounds, a comparison that would obviously not be possible for any loci genotyped in one parent but not the other as a result of variation in sequencing coverage. To reduce the effects of such missing data, we imported genotypes for Tsu-1 and Kas-1 from resequencing data (McKay, unpublished results) for any loci genotyped in one parent but not the other.

KASP SNP genotyping assays (LGC Genomics, Teddington, Middlesex, UK) were used for sub-NIL development. Primer sequences [see Additional file 8] were designed using sequence data from TAIR10 [71] for amplification of SNPs identified and validated on the SNPlex genotyping system (Applied Biosystems) as described in [38]. KASP is a novel allele-specific PCR assay that utilizes a FRET (Fluorescence Resonance Energy Transfer) system. In short, along with a common primer, allele-specific primers are designed to include a unique 18 bp sequence at the 5’ end. The unique sequences are identical to a pair of oligonucleotides with 3’ bound quenchers for a complement pair of 5’ fluorescently labelled oligos inside the reaction mix. During PCR, allele specific amplification leads to the generated product(s) outcompeting the quencher containing oligos for binding to the fluorescently labelled oligos, allowing for an observable signal to be measured using a light reader. The intensity of the signal(s) allows for a quantitative measure of SNP copy number.

### Estimating chromosomal introgression length and number

The physical length of introgressions in the final NIL library was estimated using graphical genotypes [44]. Physical length estimates of introgressions flanked by SSR markers were made using the location of the forward primers, SNP locations were determined by their location in the Col-0 reference genome. To avoid false-positives, an introgression was scored based on the presence of at least 3 consecutive markers with the Kas-1 genotype. Introgression boundaries were then defined by three consecutive markers with an alternative genotype. This helped avoid over-estimating introgression numbers due to occasional incorrect allele calls or differences in the location of loci in this population relative to the Col-0 genome used as a reference for mapping sequence reads. For the analysis of introgression discovery at varying marker densities an Excel Macro was written to sum the number of heterozygous and homozygous introgressions discovered. The loci included in replicated sampling were selected randomly using Excel’s RAND function.

### Candidate gene identification

The full list of genes expected to lie within the QTL interval spanning physical positions 505,086 to 5,273,972 was assembled using TAIR10 [71]. GO annotations for the full gene list were downloaded using the Bulk Data Retrieval and Analysis tool on TAIR10 and searched using the terms abscisic acid (ABA), stomata and water. Gene enrichment analysis was performed using the GO enrichment analysis tool in AmiGO [55].

## Competing interests

The authors declare that they have no competing interests.

## Authors’ contributions

SS, TEJ and JKM conceived the study. SY organized and conducted the backcrossing and selfing. SS wrote and implemented the algorithms for selecting RILs and backcross lines. EM produced the sequencing libraries and analyzed the genotype data with RSF. GR, WLB, JLM and JKM performed the stomatal conductance phenotyping experiments. RSF and JLM analyzed the quantitative genetic data. RSF wrote the manuscript with JLM and JKM. All authors read and approved the final manuscript.

## Supplementary Material

Additional file 1 R script used to select RILs for backcrossing. Click here for file

Additional file 2 R script used to select NILs for introgressions spanning the genome. Click here for file

Additional file 3 CSV Table of genotypes of the NIL population. Click here for file

Additional file 4 PDF Table summarizing the number introgressions discovered on each chromosome in the coarse and dense maps. Click here for file

Additional file 5 TIF Bar graph of mean dark conductance of RILs carrying the Kas-1 allele at the QTL on chromosome 1 relative to RILs carrying the Tsu-1 allele. Click here for file

Additional file 6 **TIF Genome-wide LOD graph of g**_**0 **_**QTL scans.** Click here for file

Additional file 7 CSV Table of candidate genes in QTL interval selected based on relevant GO terms. Click here for file

Additional file 8 CSV Table of the primer sequences used for KASP assays. Click here for file
