# Supplementary material for: Development of a next-generation NIL library in Arabidopsis thaliana for dissecting complex traits
Source: BMC Genomics. 2013 Sep 25;14:655. doi: 10.1186/1471-2164-14-655 (PMC3849958; doi:10.1186/1471-2164-14-655)
Supplement: Additional file 4 — PDF Table summarizing the number introgressions discovered on each chromosome in the coarse and dense maps. [file 1471-2164-14-655-S4.pdf]

Additional File 4 – Comparison of introgressions discovered using the dense (930 2b-RAD) and coarse (81 SSR/Sequenom) maps

Chromosome	homozygous		heterozygous		Total		Δ
	Dense	Coarse	Dense	Coarse	Dense	Coarse	
1	33	21	44	23	77	44	33
2	10	8	24	19	34	27	7
3	27	9	39	11	66	20	46
4	22	14	57	32	79	46	33
5	9	5	23	18	32	23	9
Total	101	57	187	103	288	160	128
