# Supplementary figures and images for: Development of a next-generation NIL library in Arabidopsis thaliana for dissecting complex traits
Source: BMC Genomics. 2013 Sep 25;14:655. doi: 10.1186/1471-2164-14-655 (PMC3849958; doi:10.1186/1471-2164-14-655)

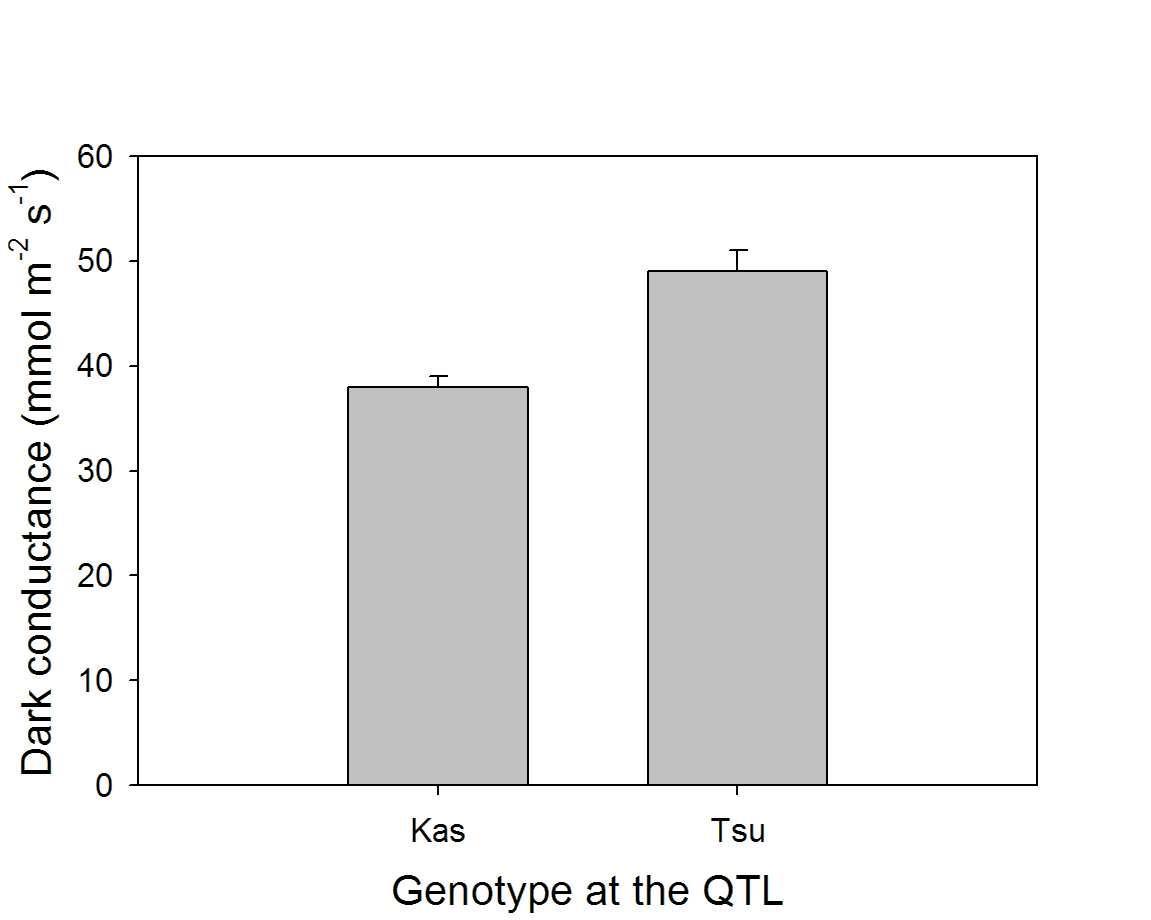

Supplement: Additional file 5 — TIF Bar graph of mean dark conductance of RILs carrying the Kas-1 allele at the QTL on chromosome 1 relative to RILs carrying the Tsu-1 allele. [file 1471-2164-14-655-S5.tiff]

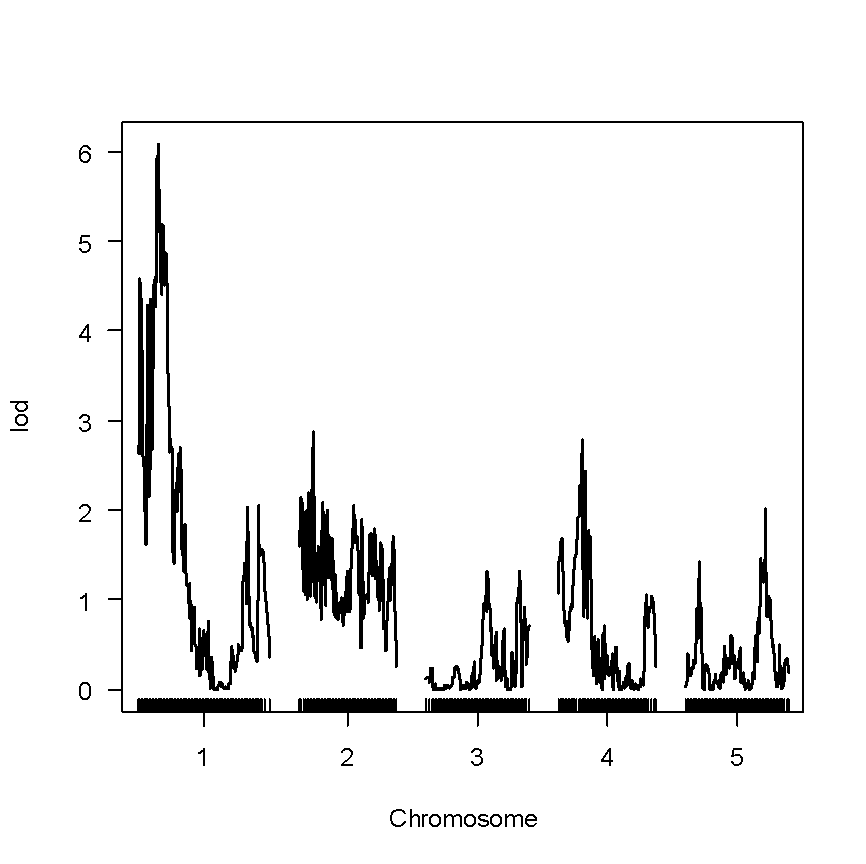

Supplement: Additional file 6 — TIF Genome-wide LOD graph of g0 QTL scans. [file 1471-2164-14-655-S6.tiff]
